# Supplementary material for: Complex‐centric proteome profiling by SEC‐SWATH‐MS
Source: Mol Syst Biol. 2019 Jan 14;15(1):e8438. doi: 10.15252/msb.20188438 (PMC6346213; doi:10.15252/msb.20188438)
Supplement: Supplementary file 7 — Dataset EV6 [file MSB-15-e8438-s007.zip › feature_plots_bioplex/P17931.pdf]

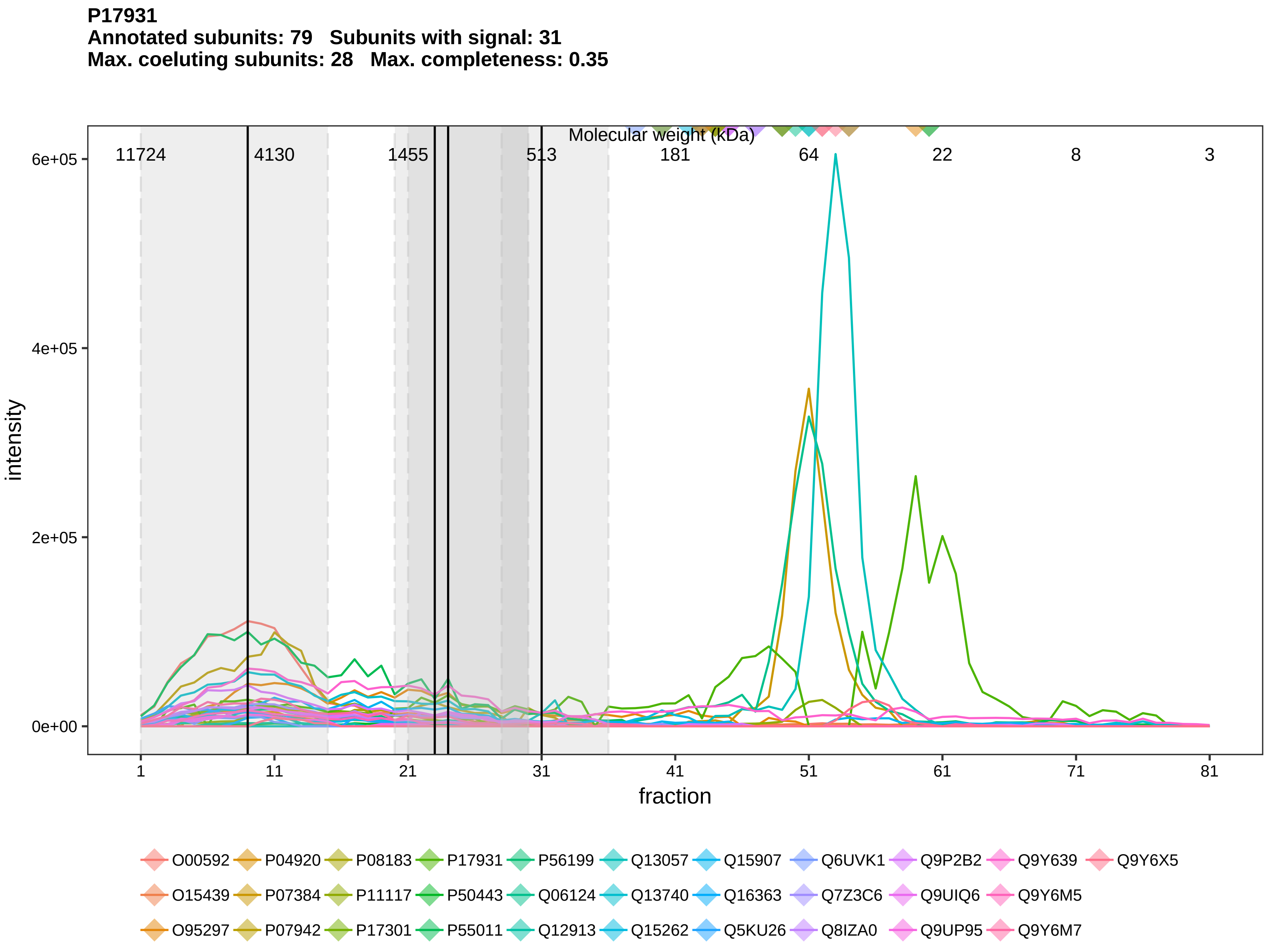
P17931
Annotated subunits: 79 Subunits with signal: 31
Max. coeluting subunits: 28 Max. completeness: 0.35
Molecular weight (kDa)
11724
4130
1455
513
181
64
22
8
3
6e+05
4e+05
2e+05
0e+00
intensity
1
11
21
31
41
51
61
71
81
fraction
O00592
P04920
P08183
P17931
P56199
Q13057
Q15907
Q6UVK1
Q9P2B2
Q9Y639
Q9Y6X5
O15439
P07384
P11117
P50443
Q06124
Q13740
Q16363
Q7Z3C6
Q9UIQ6
Q9Y6M5
O95297
P07942
P17301
P55011
Q12913
Q15262
Q5KU26
Q8IZA0
Q9UP95
Q9Y6M7
